# BRAF-mediated brain tumors in adults and children: A review and the Australian and New Zealand experience

**DOI:** 10.3389/fonc.2023.1154246

**Published:** 2023-04-14

**Authors:** Sarah M. Trinder, Campbell McKay, Phoebe Power, Monique Topp, Bosco Chan, Santosh Valvi, Geoffrey McCowage, Dinisha Govender, Maria Kirby, David S. Ziegler, Neevika Manoharan, Tim Hassall, Stewart Kellie, John Heath, Frank Alvaro, Paul Wood, Stephen Laughton, Karen Tsui, Andrew Dodgshun, David D. Eisenstat, Raelene Endersby, Stephen J. Luen, Eng-Siew Koh, Hao-Wen Sim, Benjamin Kong, Nicholas G. Gottardo, James R. Whittle, Dong-Anh Khuong-Quang, Jordan R. Hansford

**Affiliations:** ^1^ Department of Paediatric and Adolescent Oncology/Haematology, Perth Children’s Hospital, Nedlands, WA, Australia; ^2^ Children’s Cancer Centre, Royal Children’s Hospital, Melbourne, VIC, Australia; ^3^ Sydney Children’s Hospital, Children’s Cancer Institute, University of New South Wales, Randwick, NSW, Australia; ^4^ School of Women’s and Children’s Health, University of New South Wales, Randwick, NSW, Australia; ^5^ Department of Medical Oncology, Peter MacCallum Cancer Center, Melbourne, VIC, Australia; ^6^ Michael Rice Cancer Centre, Women’s and Children’s Hospital, North Adelaide, SA, Australia; ^7^ Department of Oncology, Children’s Hospital at Westmead, Sydney, NSW, Australia; ^8^ Australasian Children’s Cancer Trials, Clayton, VIC, Australia; ^9^ Children’s Cancer Institute, Lowy Cancer Research Centre, University of New South Wales (UNSW) Sydney, Sydney, NSW, Australia; ^10^ School of Clinical Medicine, University of New South Wales (UNSW) Medicine and Health, University of New South Wales (UNSW) Sydney, Sydney, NSW, Australia; ^11^ Queensland Children’s Hospital, University of Queensland, Brisbane, QLD, Australia; ^12^ Westmead Children’s Hospital, University of Sydney, Westmead, NSW, Australia; ^13^ Department of Pediatric Oncology, Royal Hobart Hospital, Hobart, TAS, Australia; ^14^ Department of Pediatric Oncology, John Hunter Children's Hospital, Newcastle, NSW, Australia; ^15^ Monash Medical Centre, Peter MacCallum Cancer Centre, Melbourne, VIC, Australia; ^16^ Starship Blood and Cancer Centre, Starship Children’s Hospital, Auckland, New Zealand; ^17^ Children’s Haematology/Oncology Centre, Christchurch Hospital, Christchurch, New Zealand; ^18^ Murdoch Children’s Research Institute, Melbourne, VIC, Australia; ^19^ Department of Paediatrics, University of Melbourne, Melbourne, VIC, Australia; ^20^ Brain Tumour Research Program, Telethon Kids Cancer Centre, Telethon Kids Institute, Nedlands, WA, Australia; ^21^ Centre for Child Health Research, University of Western Australia, Perth, WA, Australia; ^22^ Sir Peter MacCallum Department of Oncology, The University of Melbourne, Melbourne, VIC, Australia; ^23^ Department of Radiation Oncology, Liverpool and Macarther Cancer Therapy Centres, Liverpool, NSW, Australia; ^24^ Department of Medicine, University of New South Wales, Sydney, NSW, Australia; ^25^ Ingham Institute for Applied Medical Research, Liverpool, NSW, Australia; ^26^ National Health and Medical Research Council (NHMRC) Clinical Trials Centre, University of Sydney, Sydney, NSW, Australia; ^27^ School of Clinical Medicine, Faculty of Medicine and Health, University of New South Wales, Sydney, NSW, Australia; ^28^ Department of Medical Oncology, The Kinghorn Cancer Centre, Sydney, NSW, Australia; ^29^ Department of Medical Oncology, Chris O’Brien Lifehouse, Sydney, NSW, Australia; ^30^ Department of Medical Oncology, Royal North Shore Hospital, St Leonards, NSW, Australia; ^31^ Personalised Oncology Division, Walter and Eliza Hall Institute of Medical Research, Parkville, VIC, Australia; ^32^ South Australian Health and Medical Research Institute South Australia, Adelaide, SA, Australia; ^33^ South Australia ImmunoGENomics Cancer Institute, University of Adelaide, Adelaide, SA, Australia

**Keywords:** glioma, gliomagenesis, MAPK signaling, BRAF, BRAF inhibitors, access

## Abstract

The mitogen-activated protein kinase (MAPK) pathway signaling pathway is one of the most commonly mutated pathways in human cancers. In particular, BRAF alterations result in constitutive activation of the rapidly accelerating fibrosarcoma–extracellular signal–regulated kinase–MAPK significant pathway, leading to cellular proliferation, survival, and dedifferentiation. The role of BRAF mutations in oncogenesis and tumorigenesis has spurred the development of targeted agents, which have been successful in treating many adult cancers. Despite advances in other cancer types, the morbidity and survival outcomes of patients with glioma have remained relatively stagnant. Recently, there has been recognition that MAPK dysregulation is almost universally present in paediatric and adult gliomas. These findings, accompanying broad molecular characterization of gliomas, has aided prognostication and offered opportunities for clinical trials testing targeted agents. The use of targeted therapies in this disease represents a paradigm shift, although the biochemical complexities has resulted in unexpected challenges in the development of effective BRAF inhibitors. Despite these challenges, there are promising data to support the use of BRAF inhibitors alone and in combination with MEK inhibitors for patients with both low-grade and high-grade glioma across age groups. Safety and efficacy data demonstrate that many of the toxicities of these targeted agents are tolerable while offering objective responses. Newer clinical trials will examine the use of these therapies in the upfront setting. Appropriate duration of therapy and durability of response remains unclear in the glioma patient cohort. Longitudinal efficacy and toxicity data are needed. Furthermore, access to these medications remains challenging outside of clinical trials in Australia and New Zealand. Compassionate access is limited, and advocacy for mechanism of action-based drug approval is ongoing.

## 1 Introduction

Cancer is characterized by acquired genetic changes impacting on signaling pathways and driving tumor growth, evolution, and resistance to treatment. These pathways involve the interaction of proteins with “switch-like” activation or inhibition of downstream factors under genomic, transcriptomic, and epigenetic control. Large-scale, high-throughput genomic sequencing has led to an improved understanding of molecular pathways in tumorigenesis with the emergence of precision medicine and targeted therapies changing the outcomes for patients with many solid cancers (1, 2). In contrast, treatment options and survival for both pediatric and adult patients with glioma, the most common form of primary brain cancer, have remained stagnant for decades (3–5).

The mitogen-activated protein kinase (MAPK) pathway is one such complex signaling cascade that is frequently dysregulated in cancer and implicated in oncogenesis, tumor progression, and resistance to treatment (6). Of particular relevance are the rapidly accelerating fibrosarcoma (RAF) family of serine/threonine kinases that are commonly mutated in many human cancer types including colorectal, thyroid, and non–small cell lung cancers (NSCLC), melanoma, and gliomas (7, 8). Activating mutations and fusions of *BRAF* result in constitutive activation of the RAF–mitogen-activated extracellular signal–regulated kinase (MEK)–MAPK signaling pathway, leading to cell proliferation and survival. Discovery of the oncogenic capacity of *BRAF* spurred the development of RAF inhibitors, with proven clinical benefit in *BRAF* aberrant melanoma, colorectal cancer, NSCLC, and papillary thyroid carcinoma (9–12). These successes have prompted further understanding of the role of *BRAF* alterations in tumorigenesis and as a therapeutic target in gliomas.

Gliomas are a heterogeneous group of primary brain tumors. Although rare, accounting for <2% of all new cancers (13, 14), gliomas are among the most lethal, particularly in children and young adults (15). In addition, gliomas lead to significant morbidity (16) and account for a disproportionate impact on the healthcare system. Historically, gliomas have been divided into four grades based on morphology, grouped into low-grade glioma (LGG) (grades 1 and 2) and high-grade glioma (HGG) (grades 3 and 4). Although there are features common to both pediatric and adult gliomas, important differences with respect to epidemiology (17), genomic changes (17), and the role of neurodevelopment (18) impact treatment decisions. For example, grade 1 tumors, such as pilocytic astrocytoma (PA), almost exclusively occur in children and young adults and, if completely resected, are usually cured with surgery alone (19). In contrast, grade 2–4 tumors, which represent the dominant grades in adults, are characterized by diffuse infiltration, making a gross total resection (GTR) unlikely in the majority of neuroanatomic locations, rendering them nearly impossible to cure (20).

More recent genomic classification has advanced the prognostic impact of molecular diagnosis (21). The recently updated World Health Organization (WHO) classification of central nervous system (CNS) tumors has further integrated molecular features into the diagnosis, paving the way for the investigation of targeted therapeutic strategies, which have the potential to improve outcomes for these patients (21). MAPK pathway alterations have been identified across glioma subtypes, ages, and grades spurring several clinical trials (22–29) following the success of *BRAF* inhibitors in melanoma (30). Early studies of *BRAF* inhibitors as monotherapy (23) and, more recently, in combination treatment (22) have shown encouraging results in gliomas. In addition, newer RAF dimer inhibitors and combination therapies are currently under investigation, although access to treatment outside of clinical trials remains challenging.

Despite growing evidence of benefit, multiple challenges impact the successful development of MAPK targeting drugs in glioma. Notably, currently targetable genetic alterations are infrequently observed in CNS tumors (31). In addition, the permeability of the blood–brain barrier (BBB) (32) and tumor heterogeneity are potential limitations to the impact of many of these targeted therapeutics. These challenges have required a rethinking of trial design and mechanisms of drug approval, which are common to rare cancers (33, 34). In addition, there are several unanswered questions and controversies in the field, with respect to treatment duration (35), management of toxicity (36), and the potential for paradoxical activation of signaling pathways with treatment, leading to malignant transformation (37). This review details the status of MAPK pathway targeting in pediatrics and adult gliomas, highlighting its role in clinical practice, particularly in Australia and New Zealand, and identifying ongoing research questions for the field.

## 2 Biology of ERK/MAPK signaling and specific alterations

### 2.1 MAPK signaling pathway in normal brain

The MAPK pathway consists of integral groups of signal transduction molecules responsible for the fine-tuned regulation of key cellular processes that have been heavily implicated in tumorigenesis. The MAPK pathway is activated in most regions of the brain and involved in key neurodevelopmental processes (38). There are three distinct subfamilies of MAPK signaling in humans. These are the extracellular signal–regulated kinase (ERK) pathway, the c-Jun N-terminal kinase pathway, and the p38 pathway. The ERK/MAPK pathway is the key pathway dysregulated in gliomas and, therefore, will be the focus of this review.

**Figure 1:**
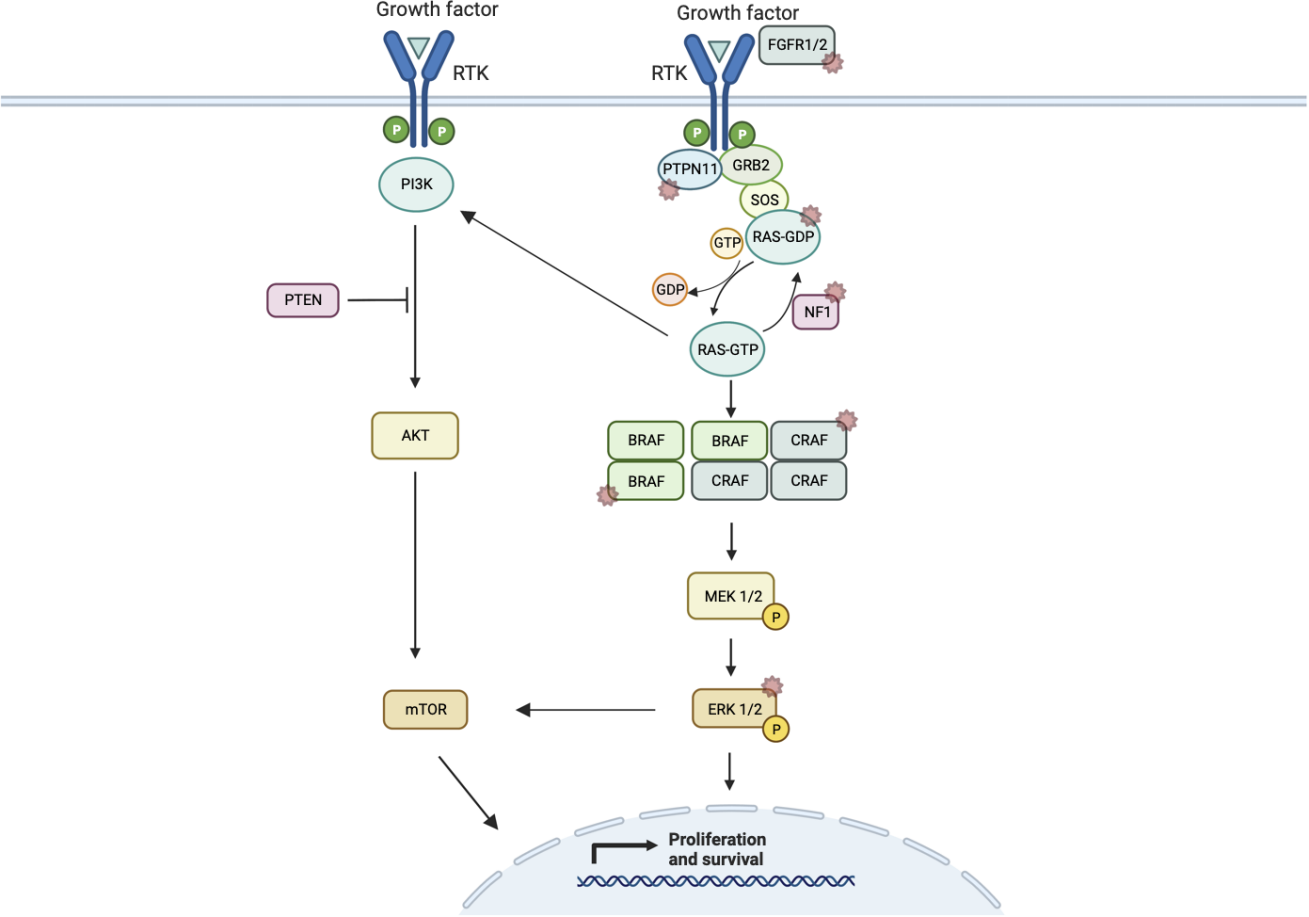
Schematic representation of ERK/MAPK pathway signaling showing normal dimerization of MAP3Ks and cross-signaling of the PI3K/AKT/mTOR pathway. Starred pathways indicate known alterations implicated in cancer.

Under normal circumstances, the ERK/MAPK pathway is triggered by ligand-mediated activation of receptor tyrosine kinases (RTKs) (for example, FGFR1), which triggers guanine nucleotide-binding protein (GTPase) belonging to the r**a**t sarcoma virus (RAS) family [e.g., Harvey rat sarcoma virus (HRAS), Kirsten rat sarcoma virus (KRAS), and Neuroblastoma rat sarcoma virus (NRAS)] (39). RAS is switched off by GTPAse activated proteins including neurofibromin 1 (*NF1*). Following activation, RAS, in turn, recruits members of the RAF family of serine/threonine kinases to the plasma membrane that induces dimerization and activation (**Figure 1**). RAS has an active guanosine triphosphate (GTP)-binding conformation and an inactive guanine diphosphate (GDP)-binding confirmation. Binding of extracellular signals to the receptor induces binding of growth factor receptor-binding protein 2 (Grb2) to the activated receptor and autophosphorylation at the C-terminus region of son of sevenless (SOS) to form the GrB2–SOS complex. This, in turn, drives activation of SOS and Ras-GDP to replace GDP with GTP, thereby activating the RAS pathway (40). In this instance, RAF acts as a MAPK kinase (MAP3K) with three different subtypes—ARAF, BRAF, and CRAF, of which BRAF is the most active (41). Through downstream phosphorylation, they then activate MEK1/2 (biologically equivalent MAPK kinases) before finally activating ERK1/2 (MAPKs). In the nucleus, ERK1/2 transcriptionally regulates genes involved in proliferation and cell survival, including cAMP response element–binding protein, as well as transcriptional regulator Myc-like (c-Myc) and nuclear factor kappa B (39).

### 2.2 MAPK pathway alterations in cancer

Mutations in the MAPK pathway are the most common molecular alterations in cancer (42). These include activating mutations or in-frame fusions in components of the signaling cascade or loss-of-function mutations in negative regulators, leading to constitutive activation of the MAPK pathway (39). Since their discovery, they have provided an appealing target for directed therapies not only due to their prevalence but also due to their recognized role as the genetic drivers in malignancy. In more recent times, dysregulation of this pathway has been found to be almost universally present in glioma (43–45) and, particularly, all types of pediatric LGG (pLGG) (44).

#### 2.2.1 BRAF alterations

The *BRAF* gene located at chromosome 7q34 encodes for the kinase BRAF. Under normal cellular conditions, it is regulated by the N-terminal autoinhibitory domain binding to its catalytic domain, resulting inhibition of BRAF. This process is blocked by RAS activation. Mutations in V-RAF and its human ortholog, BRAF, were the first implicated in cancer, with hotspot mutations in V600 codons, demonstrating their oncogenic potential through the transformation of NIH3T3 cells (46). *BRAF* alterations lead to constitutive MAPK signal pathway activation, bypassing the need for proliferative signals and promoting cellular proliferation, survival, and dedifferentiation (**Figure 1**). Activating mutations of *BRAF* can occur as point mutations, in-frame deletions, or fusions with other kinases. To date, more than 30 *BRAF* alterations have been associated with human cancers and are grouped according to kinase activity (47), which suggests mutation class also implies varying sensitivity to *BRAF* inhibitors.

Class 1 mutations (kinase-activated, codon 600) signal as RAS-independent active monomer (48). This group results in strong activation of BRAF kinase activity and constitutive activation of MAPK pathway. MAPK activation leads to negative feedback on RAS preventing BRAF dimerization and allowing BRAF-mutant proteins to signal as monomers (42–49). The most common class 1 mutation is V600E, which occurs due to a single-nucleotide substitution mutation at position 1799T>A, resulting in replacement of valine (V) with glutamic acid (E) at codon 600. The other class 1 mutations, including V600D, V600K, and V600R, are less common, and their clinical impact, particularly in glioma, is largely unclear.

**Figure 2:**
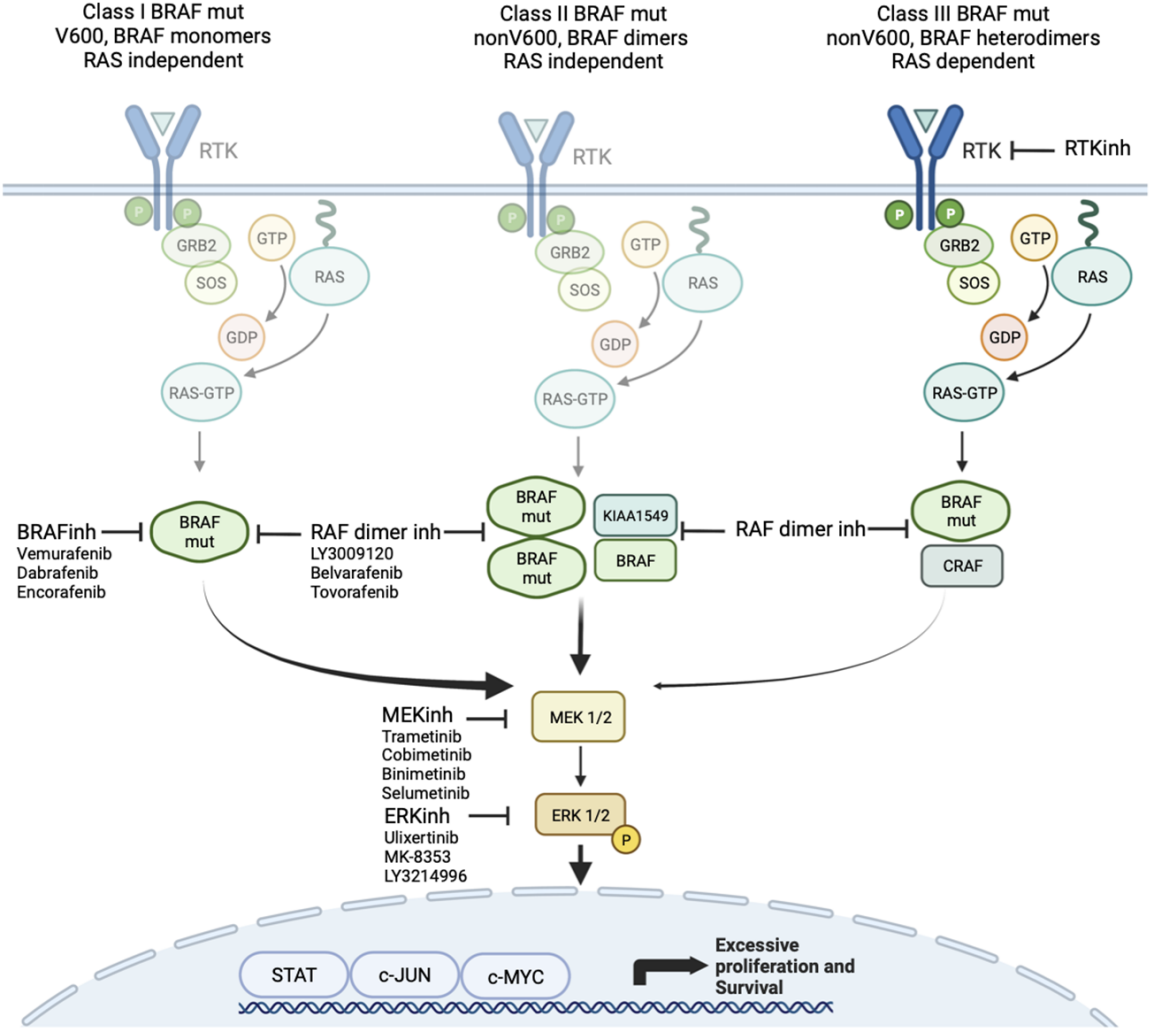
Targeted agents focused on MAPK signaling including class 1 and 2 BRAF inhibitors and MEK and ERK inhibitors.

Class 2 mutations (kinase-activated, non-codon 600) include point mutations and fusions that lead to RAS-independent activation of MEK (**Figure 2**). Commonly described class 2 point mutations occur in the activation segment and include *K601E/N/T*, *L597Q/C*, and *G469A/V/R*. These mutations typically result in less pathway activation in comparison to *BRAF* V600 mutants (48). The *KIAA1549-BRAF* fusion is most common in pLGG and results in replacement of the N-terminal auto-regulatory domain with the fusion component of *KIAA1549*, and, consequently, this new fusion protein is constitutively active. This has been confirmed to cause activation of the MAPK pathway with high levels of phosphorylated ERK production *in vitro (* 50). Multiple *KIAA1549-BRAF* fusions have been described including 16;9, 15;9, 16;11, 18;10, and 19;9, all resulting in the loss of *BRAF*’s regulatory domain (51).

Class 3 mutations (kinase-impaired) enhance MAPK signaling through RAS. These include those within the P-loop, catalytic loop, and Asp-Phe-Gly (DFG) motif (52). These mutations cause impaired kinase activity and are classed as kinase “dead”. They are sensitive to MAPK pathway–mediated feedback, and their activation of signaling is RAS-dependent. The mutant BRAF bind more tightly than wild-type BRAF to RAS-GTP, and their binding to and activation of wild-type CRAF is enhanced, leading to increased MAPK -signaling. In addition to the more well described class 1 to 3 mutations, many other mutations and fusions have been reported where the effect on kinase function is not well understood.

#### 2.2.2 Other ERK/MAPK pathway alterations

Although *BRAF* has been subject to much interest in gliomas, multiple other pathway alterations are observed across cancer subtypes, with relevance to treatment. These include mutations in *NF1* and RAS family genes, as well as infrequent mutations in *MEK* and *ERK*. Moreover, mutations can occur in the genes that encode RTKs including *FGFR1*, *EGFR*, *NTRK1*, and *cMET*. In addition, MAPK signaling involves extensive regulatory cross talk with other relevant pathways such as phosphoinositide-3-kinase/v-akt murine thymoma viral oncogene homolog 1/mechanistic target of rapamycin kinase (PI3K/AKT/mTOR) and transforming growth factor beta signaling pathways, with implications for treatment and resistance.

The ***NF1*** gene encodes the RAS GTPase activating protein neurofibromin with mutations, leading to loss of function of neurofibromin associated with activation of RAS/MAPK and PI3K/AKT signaling in cancer (53). It acts as a negative regulator of RAS, the absence of which leads to inappropriate upregulation of MAPK pathway and increased cell proliferation. Neurofibromatosis type 1 (NF1) is a common autosomal dominant genetic disorder characterized by mutations in *NF1*, associated with almost universal development of cutaneous neurofibromas, as well as plexiform neurofibromas, optic pathway glioma (OPG), and malignant peripheral nerve sheath tumors. Somatic mutations in *NF1* also occur in 5% to 10% of human cancers, particularly lung cancer (54), glioblastoma (45), and breast cancer (55), and are associated with resistance to therapy. Mutations in other genes that impact the RAS/MAPK pathways manifest as RASopathies such as Noonan and Costello syndrome. These syndromes exhibit multiple overlapping phenotypic features to NF1 including increased cancer risk (56).

Although mutations in other RAS family genes (e.g., *KRAS* and *NRAS*) are observed in up to 20% of all cancers (57), particularly lung (0.2% to 32%), melanoma (1.2% to 17%), and colorectal cancer (33% to 50%), they are infrequent in glioma and not associated with any particular phenotype (58). In addition, whereas upstream mutations in RAS and RAF are common, mutations in ERK and MEK are exceptionally rare. Nevertheless, they represent an important target as downstream regulators of innate and acquired resistance to RAF inhibitors.

Complementary signaling pathways, such as PI3K/AKT/mTOR, converge on transcription factors that alter gene expression, interacting with ERK/MAPK family members to influence signal transduction in cancer. Notably, the PI3K/AKT/mTOR pathway can also be activated *via* RTKs and RAS, resulting in protein synthesis that sustains cell growth, leading to a dynamic interaction between RAS/ERK and RAS/PI3K (59) (**Figure 1**). Another consideration is the cross-inhibition between MAPK and PI3K/AKT, for instance, when activated AKT phosphorylates a highly conserved serine residue in RAF regulatory domain, leading to its inactivation and therefore inhibiting MAPK signaling (60). Interestingly, these two pathways can also cross-activate each other *via* ERK1/2. Mutations in *PTEN* influence response inhibitors of MEK and mTOR (61). This cross talk between key signaling pathways is an emerging focus of modern clinical trials (62, 63).

## 3 The genomic landscape of pediatric and adult glioma

### 3.1 Classification

Gliomas are a heterogenous group of primary brain cancers occurring at any age, which arise from cells of glial lineage and resemble astrocytes (astrocytoma), ependymal cells (ependymoma), and oligodendrocytes (oligodendroglioma). The WHO classification system provides a “malignancy” range, based on the natural history of gliomas from grade 1 (relatively benign) to grade 4 (malignant), which is broadly grouped into LGG (grades 1 and 2) and HGG (grades 3 and 4). However, despite similar histological appearances, tumors often harbor different molecular features that contribute to patient outcome. As such, in 2016, the WHO CNS revised fourth edition (64), moved beyond histologic classification, informed by the Harlem Consensus Guidelines, and incorporated molecular findings into diagnosis. In 2021, the WHO CNS fifth edition (21) built on the revised fourth edition, aided by the Consortium to Inform Molecular and Practical Approaches to CNS Tumor Taxonomy – Not Officially WHO (cIMPACT-NOW) (65), and further incorporated molecular diagnostics, with molecular features explicitly required for diagnosis and in some cases overriding histological features. Classification of glioma is an evolving process with much still to be learned about tumor phenotypes and biological behavior, but integrating molecular classification allows a framework in which to progress our understanding and has laid the foundation for precision treatment strategies to be investigated.

Historically, pediatric gliomas were classified on the basis of the same criteria designed for adult glioma and have long been considered to have similar biology. Emphasizing that “kids are not little adults”, while pediatric gliomas may share histological similarities with adult gliomas, they differ significantly in prevalence, genetics, biology, and prognosis (17). Thus, it is essential to distinguish between the two entities to better tailor care. Recognizing this, the WHO CNS fifth edition reclassified diffuse gliomas into adult type and pediatric type (21). Their classification does not depend on age but on representative molecular alterations, and, indeed, pediatric-type gliomas can occur in adults and *vice versa*.

Understanding the correlation between the common molecular drivers and histologically diverse disease represents an evolving challenge. This is particularly the case in pLGG where histologic lesions may not always reliably predict progression and prognosis (44). The recognition of the importance in identification and classification of these genetic drivers is reflected in the current paradigm shift of classification of pLGGs with new “hybrid taxonomy” being introduced in the WHO 2021 classification of CNS tumors (21). Notably, there are three newly described diffuse pLGGs, two of which are defined by their molecular drivers (diffuse astrocytoma, MYB or MYBL1-altered and diffuse LGG, and MAPK pathway–altered).

In addition to mutation and copy number changes, the past decade has seen efforts in using methylation arrays to refine CNS tumor classification (66–70), and methylation arrays are increasingly incorporated into comprehensive molecular profiling and precision medicine programs. Although caveats remain on both availability and optimal methodology, DNA methylation profiling can provide valuable insights into cell heterogeneity and the aberrant epigenetic processes that alter chromatin modeling and gene regulation, resulting in cancer. Indeed, the four classes of pLGG segregate into reasonably distinct methylation clusters, despite sharing similar drivers, including MAPK pathway activation (21). With the increasing use of these technologies, the subgroups of glioma are likely to continue to expand on the complexity of diagnosis.

### 3.2 Epidemiology and prognosis

CNS tumors are the most frequent solid cancers in children and adolescents aged 15–19 years old and the most common cause of cancer related death in children and adolescents and young adults (AYAs) (14). Among pediatric CNS tumors, 50% are gliomas (13), with most presenting as WHO grade 1 and, rarely, slower growing grade 2 pLGGs. Pediatric HGGs account for 10% of brain tumors in children and, despite surgery and adjuvant therapy, unfortunately, 70% to 90% of affected children die within 2 years of diagnosis (71). In adults, in contrast, gliomas are the commonest primary intracranial tumor and are predominantly high grade. Age also influences prognosis within groups, with elderly patients with HGG having the poorest survival (72) and children under age 3 having the best outcome (73, 74).

Within LGG, PAs are common in children and WHO grade 2 diffuse astrocytomas are rare. In young adulthood, a transition occurs, with diffuse glioma becoming more common. Similarly, the risk of malignant transformation (i.e., progression from LGG to HGG) is significantly less common in pLGG (75) compared to adults with LGG (76, 77). These features highlight a critical difference between age groups and pathology. Whereas the subsets of pLGG, particularly PA, can be cured, the diffuse infiltrating pattern of adult gliomas is nearly impossible to cure using a traditional approach. Thus, disease biology dictates a requirement for different treatment approaches that considers the underlying molecular lesion.

### 3.3 Biological differences between pediatric and adult glioma

#### 3.3.1 Low-grade glioma

pLGGs are generally more indolent, have fewer genetic drivers, and are more genetically homogeneous than adult gliomas, presenting significant therapeutic potential (78). The most common subtype of pLGG, PA (WHO grade 1), accounts for 65% of tumors and is defined by alteration of the MAPK pathway, which is further detailed below. In contrast, adult LGG (aLGG) are mostly diffuse WHO grade 2 gliomas, with peak incidence at 35–40 years, defined by isocitrate dehydrogenase *(IDH)* mutations and *ATRX* mutations in astrocytic tumors, and *IDH* mutations and 1p19q co-deletion in oligodendroglial tumors. *IDH* mutations are almost absent in children (44). pLGGs are also more likely to be associated with a genetic predisposition, such as NF1 syndrome and tuberous sclerosis complex (79, 80).

In children, PAs are considered relatively slow growing lesions with 10-year survival of over 90%. Many only require surgery and very infrequently progress to higher-grade lesions (81, 82). However, population-based studies identified that there is declining 5-year survival with age from 95% in pediatric patients to 92.3% ages 20–39, 78.6% in ages 40–59, and 63.7% ages >60 years (83). This may be attributable to more frequent *KIAA-BRAF* fusions in children, associated with improved survival, and potential misdiagnosis of H3K27M diffuse midline glioma as PA in adults (84). In comparison to more circumscribed PAs, WHO grade 2 gliomas have a diffuse infiltrative pattern by nature. This decreases the capacity for GTR and thus increases the risk of progression following surgery (85).

Anatomically most pLGGs occur in the cerebellum, with some pathognomonic locations according to mutation. Higher rates of fusion positive LGG occur in the cerebellum and mutation-driven disease in the supratentorium (44). In comparison, aLGGs classically occur supratentorially, often in eloquent regions of brain with implications for the ability to achieve maximal surgical resection. Finally, although pLGG may involve leptomeninges and spinal metastasis (86), this is almost never encountered in aLGG.

#### 3.3.2 High-grade glioma

In the last decade, there has been an explosion of discovery accompanied by opportunity around development and introduction of targeted therapy for glioma. Much hope was placed on targeting mutations in HGG, given the exceptionally poor outcomes of patients with this disease (87, 88). Excitement grew when early reports showed *BRAF* mutations occurred in glioma, given the successes of targeting this molecule in other diseases (89, 90). Although our understanding has grown, most gliomas in pediatric and adult settings are not yet targetable with histone variants primarily responsible for most pediatric gliomas (91). In the largest pHGG cohort reported to date, only 6% of pHGGs were found to carry *BRAF* V600E alterations (91). This frequency drops as age at presentation increases. In several large retrospective series, aHGG with *BRAF* alterations was even rarer, with only 4 of 254 (92) and 4 of 387 (93), equating to a frequency of only 1.2%. Interestingly, in both series, atypical histology, including epithelioid, gliosarcoma, and giant cell variants, was more likely to contain *BRAF* V600E changes.

### 3.4 Malignant transformation

Unlike aLGG, pLGG rarely transforms to a highly malignant histopathological phenotype. Pediatric studies demonstrate malignant progression from pLGG to pHGG in 2.9% to 11% of patients (75, 94, 95). Mistry et al. (95) and Lassaletta et al. (96) have demonstrated a high incidence of malignant progression in pLGG with *BRAF* V600E mutations, particularly in combination with *CDKN2A* biallelic deletion. In more recent studies, *pTERT* mutations predict poor outcome in DNA methylation defined pleomorphic xanthoastrocytomas (PXA) and are a more robust indicator of risk of transformation (97).

Traditionally, up to 70% of aLGGs progress to HGG (98). However, using modern risk stratification and molecular characterization, this risk is now considered to be much lower (77). In a study of 486 adults with molecularly characterized and risk-stratified aLGG, malignant transformation was observed in 84 patients (17%) for the entire cohort. Little difference was seen with *IDH* status (mutated, 51 of 284 patients, 17.9%; wild type, 33 of 185 patients, 17.8%).

### 3.5 Standard of care treatment

Surgery, where feasible, is the mainstay of treatment across all ages and subgroups of disease. Surgery may be indicated to relieve mass effect, for large symptomatic lesions, improve survival, or obtain a biopsy for histological and molecular diagnosis. Highlighting the central role of surgery across most ages and subtypes, the extent of surgical resection is an independent prognostic variable (99) with lower risk of recurrence with those patients achieving GTR (19). Indeed, for PA, surgery is often curative depending on the anatomic location of the tumor. However, for WHO grade 2 diffuse gliomas and HGGs, post-operative therapy is indicated to delay progression and maintain quality of life.

#### 3.5.1 Treatment of LGG across ages

The approach to LGG therapy in children is dependent on several factors including location of tumor, the patient’s age, and risk of future morbidity. As above, in those patients with a GTR, observation only is standard. Where total resection is not feasible due to location and risk to function (i.e., optic pathway, spin, and brainstem), earlier medical treatment may be needed. Even with evolving understanding the molecular basis of disease, one of several chemotherapy regimens tends to be the therapy of choice including carboplatin and vincristine combination therapy (100–102), single-agent carboplatin (103), or single-agent vinblastine (104). Upfront targeted therapy remains limited to selected clinical trials. No matter the approach, all regimens to date result in progression-free survival (PFS) outcomes of 50%. Other chemotherapy approaches tend to be more toxic and are reserved for relapse. Radiation is generally avoided given the risk of future malignant transformation.

In AYA patients, aged 15–39 years, management of LGG straddles the management approaches between adult and pediatric populations. Consistent among pediatric and adult patients, maximal safe resection should be attempted where feasible and those that undergo GTR may be safely observed (105). In those patients with residual radiographic tumor, 5-year PFS is 55%. In younger adolescents and patients whose risk of cognitive impairment from radiotherapy is substantial, maximal safe surgical resection and delay in radiotherapy may be appropriate. As age increases, risk factors for recurrence accumulate, and immediate adjuvant therapy is more strongly recommended. However, this must be balanced against the significant morbidity associated with adjuvant treatment.

#### 3.5.2 Treatment of HGG across ages

HGG in adults and children remains a therapeutic challenge with patients having a relatively poor prognosis. Only modest benefit is seen with current therapies. In general, chemotherapy has only had limited effectiveness, whereas temozolomide (TMZ) in aHGG has improved event-free survival and overall survival (OS) compared to radiotherapy alone when *MGMT* promoter is methylated. Median survival for adult patients with *IDH*–wild-type glioblastoma following the standard treatment of maximal safe resection, irradiation, and concurrent and adjuvant TMZ chemotherapy is, on average, only 14 months (5).

Novel therapeutic strategies from adult glioma research have not yielded success in pHGG, likely reflecting that the biological differences that exist between adult and pediatric HGGs. Molecular alterations in pHGG are commonly associated with histone H3 mutations, whereas *IDH* mutations, *PTEN* loss, and *EGFR* amplifications are commonly found in adult gliomas. *BRAF* V600E mutation can be found in 1% to 8% of GBM, with a higher mutation rate in patients below the age of 30 years (20%) and in 50% of the epithelioid subtype (46, 92, 93). Given the lack of therapeutic options, the presence of *BRAF* alterations represents a unique potential for targeted therapy that has, otherwise, not been successful in gliomas. In view of the poor outcomes for conventionally treated pHGG, standard upfront adjuvant therapy with *BRAF* inhibitors for the small subset of *BRAF* V600E-mutated pHGG is proposed.

### 3.6 MAPK pathway alterations in glioma

The defining role of the MAPK pathway in the pathogenesis of glioma was first suggested by the high prevalence (10% to 15%) of OPGs in patients with germline *NF1* mutation (71, 106). More recently, large-scale analysis of pLGG by whole genome, RNA sequencing, and phospho-proteomic studies have shown that up to 95% of these tumors harbor either a mutation or exhibit upregulation of the ERK/MAPK pathway (44). These initial discoveries and the rapid progression of molecular characterization over the past decade have led to establishment of the ERK/MAPK pathway as the defining molecular driver in pLGG. *BRAF* mutations and fusions are the most common alterations observed in pLGG and are valuable diagnostic markers. *BRAF* V600 point mutations occur in approximately 20% of pLGGs (96, 107). The majority of PAs exhibit *KIAA1549-BRAF* fusion resulting from *BRAF* tandem duplication (108). Conversely, *BRAF* alterations are rarely found in adult gliomas (92, 93), although activation of the MAPK is seen in 70% of GBM through amplifications or fusions in *EGFR/PDGFRA/MET/FGRF1/2/3* genes or mutations in MAPK pathway members particularly *NF1*, reported in 15% of GBM (45).

Although *BRAF* alterations and targeting are the focus of this paper, other ERK/MAPK pathway alterations are implicated in glioma, due to their common downstream effects and the possibility of combined therapeutic targets. These can largely be classified into the following: 1) those affecting RTKs—*FGFR1/2/3*, *NTRK1/2/3*, *ALK*, *ROS1*, and *PDGFRA*; 2) alterations in RAS GTPases; and 3) alterations affecting downstream cytosolic components—*PI3K*, *PTPN11*, *CRAF, MAP2K1*, *MAPK3*, and *MAPK1*. Combined together, mutations affecting these genes are seen in <15% of pLGG cases.

#### 3.6.1 Oncogenicity of BRAF alterations in glioma

The confirmation of the integral role of the ERK/MAPK pathway was established after multiple studies, demonstrating not only the high prevalence of *BRAF* mutations in PA but also their role in tumorigenesis [reviewed in (51)]. Pfister and Jones, in independent studies, identified recurrent duplications at 7q34, the locus for *BRAF*, in PA (50, 109), with strong upregulation of BRAF protein expression, suggesting a novel mechanism for tumorigenesis given the otherwise relatively bland molecular composition of PA. This was further refined using Fluorescence in situ hybridization (FISH) and a custom oligonucleotide array that identified three breakpoints that led to the description of a novel oncogenic fusion known as *KIAA1549-BRAF (* 50). This fusion causes constitutive activation of BRAF through loss of its N-terminal domain and was observed in 66% (29 of 44) of their PA cohort. The most common breakpoint identified was at *KIAA1549* exon 16 with *BRAF* exon 9 (seen in 20 cases), which has been reproduced in larger series. Since then, much work has been done in further identifying similar and related alterations, with *BRAF* mutations remaining by far the most pervasive, at over 50%.

Importantly, despite the initial reported specificity in the traditional *KIAA1549-BRAF* rearrangement for PA, these fusions have subsequently been found to occur in a variety of other tumor histologies. In a comprehensive examination of 540 pLGG, 180 canonical *KIAA1549-BRAF* fusions were identified, with a prevalence of 83% in PA, as well as ganglioglioma (4.4%), diffuse astrocytoma (2.8%), glioneuronal tumors (2.2%), and desmoplastic infantile astrocytoma (0.6%), and 6.7% in pLGG NOS (44). It is important to be aware, therefore, that these canonical fusions are not necessarily diagnostic of a given tumor type and are tumor agnostic. In parallel, there have been other *BRAF* fusion partners identified, including *FAM131B* (110), *RNF130*, and *CLCN6* (111), which are more frequently seen in a cohort of older children with LGG affecting hemispheric or brainstem areas, contrasting with their typical predominance in the cerebellum. These rearrangements involving other fusion partners also result in the removal of BRAF’s N-regulatory domain, leading to subsequent constitutive activation of the ERK/MAPK pathway as seen with the canonical fusion. Whether these unique clinical features are related to a different mechanism of tumorigenesis remains to be shown.

The other prominent alteration in pLGG is *BRAF* V600E mutations. This well-known oncogenic class 1 mutation has been identified and targeted in adult malignancies, but with a wide range of clinical features and behaviors (112). These differences are likely related to other molecular features including mutational burden (113). Rarely, *BRAF* fusions and single-nucleotide mutation may occur concurrently, in 1% to 3% of pLGGs including PA and PXA (110, 114).

The distribution of *BRAF* alterations (most commonly the V600E variant) in adult brain tumors spans across biologically and clinically diverse entities and may have prognostic and therapeutic implications. *BRAF* V600E mutations, although rare (2% to 8%), can be detected in all grades of adult infiltrative gliomas (45). These patients are observed to be younger and survive relatively longer compared to *EGFR*-mutant GBM, which is putatively activated in 45% of cases. Gain-of-function mutations in *EGFR* and *BRAF* genes are thought to be mutually exclusive.

The prognostic significance of *BRAF* V600E mutation and *KIAA1549-BRAF* fusion appears to be dependent on the histological type of the primary brain tumor, the age of diagnosis, and the tumor location. *BRAF* V600E mutation usually carries a relatively more favorable prognosis in PXA but is a negative prognostic marker in gangliogliomas (115) and diencephalic pLGG (116). The *KIAA1549-BRAF* fusion tends to be associated with markedly improved outcomes in children with astrocytomas (117).

#### 3.6.2 Cellular senescence

Cellular senescence is defined by the irreversible arrest of cell division, and several investigators have observed that it can be triggered *in vivo* by mutations in *BRAF*, leading to the rationale that oncogene-induced senescence (OIS) is a mechanism of tumor suppression that restricts the progression of benign tumors (118, 119). In clinical practice, *KIAA1549-BRAF* rearrangements tend to occur in younger patients and appear to promote tumorigenesis in a dose-dependent way depending on an appropriate neurodevelopmental context (120). Interestingly, preclinical studies have shown that deregulated BRAF activity leads to increased proliferation in region-specific mouse neural stem cells but is insufficient to do so in mature astrocytes (121). Conversely, other studies have shown BRAF induction in human PA-associated glioma stem cells to lead to OIS. This dichotomy is likely related to a “dose-dependent” upregulation of the MAPK pathway and that this balance of regulation may provide a target for therapy in trying to drive tumors toward senescence (118, 119).

#### 3.6.3 Other MAPK alterations

After *BRAF* alterations, *NF1* mutations are the next common MAPK changes, at approximately 15% of all gliomas (44). Changes to *NF1*, both somatic and constitutional, result in loss of RAS suppression (**Figure 1**) and in increased MAPK signaling.

Frequent alterations in RTKs have been reported in both pediatric and adult gliomas, with FGFR being the main group of RTK affected. This includes in pLGG, fusions, tyrosine kinase duplications, or hotspot mutations in FGFR1/2, resulting in the autophosphorylation of the tyrosine kinase domain and constitutive activation independent of ligand (111). These tumors also tend to occur outside the cerebellum and in the midline (111). In aLGG, the profile of FGFR alterations is different. In a Chinese series of 993 adult glioma cases, up to 9% of patients were found to have FGFR variants, mostly amplification of *FGFR1*, whereas more fusion events were seen with *FGFR3 (* 122). FGFR variants were also more common in *IDH*–wild-type than in *IDH*-mutant gliomas.

Recently, it has been appreciated that fusions of *NTRK1-3* and other RTKs can drive glioma through MAPK, particularly in young children (73, 74). Many of these fusions have effective inhibitors that have been taken to clinical trials with some success, including in pHGG (123). Mutations and alterations affecting downstream cytosolic components, including PTPN11, ERK, and MEK, are less common. Molecular genetic changes in brain tumors are summarized in **Tables 1A**, **B**.

**Table 1A:** MAPK pathway alterations by tumor type.

	Pediatric-type diffuse low-grade gliomas		Circumscribed astrocytic gliomas		
MAPK pathway alteration	Polymorphous low-grade neuroepithelial tumor of the young (124)	Diffuse low-grade glioma, MAPK pathway–altered (21)	Pilocytic astrocytoma (21)	High-grade astrocytoma with piloid features (125)	Pleomorphic xanthoastrocytoma (21)
FGFR1/2 mutation		+	<5%	17%	
FGFR1/2 fusion	45%	++	<5%	2%	
NTRK1/2/3 fusion		+	2%		+
MET fusion		+	Single cases		
ROS1 fusion			Single cases		
KRAS SNV			Single cases	3%	
NF1 loss			10%–15%	30%	+
BRAF V600E	55%	++	5%–10%	1%	60%–80%
Other BRAF SNV					+
KIAA1549-BRAF fusion		++	>60%	20%	
Other BRAF fusion			<5%		+
CRAF fusion			Single cases		+
ERK/MAP2K1 SNV		+			

+, reported cases; ++, >20%.

**Table 1B:** MAPK pathway alterations by tumor type continued.

	Glioneuronal and neuronal tumors			
MAPK pathway alteration	Ganglioglioma (126)	Desmoplastic infantile ganglioglioma/astrocytoma (127, 128)	Diffuse leptomeningeal glioneuronal tumor (21, 129, 130)	Multinodular and vacuolating neuronal tumor (21)
FGFR1/2 mutation	5%		+	
FGFR1/2 fusion	7.5%			+
NTRK1/2/3 fusion		6%	+	
MET fusion				
ROS1 fusion				
KRAS SNV	5%			
NF1 loss	2.5%			
BRAF V600E	45%	30%	+	
Other BRAF SNV	12.5%	30%		+
KIAA1569::BRAF fusion	5%		72%	
Other BRAF fusion	5%			
CRAF fusion	2.5%		+	
ERK/MAP2K1 SNV				+

+, reported cases.

## 4 ERK/MAPK pathway inhibition in glioma

### 4.1 Biology of ERK/MAPK pathway inhibition

With near universal upregulation of the ERK/MAPK pathway, there is an upswing in preference for the use of targeted therapies in the treatment paradigm for pLGG. The three tiers of the MAPK pathway provide multiple proteins that may be targeted for inhibition (**Figures 1**, **2**). These broadly are RAF, MEK, and ERK, in order of their downstream phosphorylation events. Examining each in turn, through evaluation of pre-clinical studies, highlights the complexities of this biochemical pathway and the challenges faced in their inhibition. BRAF inhibition itself is highly desirable given its mutation prevalence and specificity for tumorigenesis in pLGG tumor cells.

Type I inhibitors are ATP-competitive and stabilize RAF in its active confirmation (“DFG-in”) while blocking its catalytic activity. An initial study of vemurafenib (PLX4032), a type I RAF inhibitor, demonstrated excellent efficacy against BRAF V600E–mutated melanomas (131). Subsequent clinical studies examining the use of a type I RAF inhibitors, such as sorafenib, in *KIAA1549-BRAF*–fused PAs were unfortunately met with paradoxical upregulation of the MAPK pathway and marked tumor growth (37). In this study, nine of the 11 patients (81.8%) experienced rapid progression of their tumor with a median time to progression of 2.8 months (37). This critical result was caused by the paradoxical activation of wild-type BRAF through heterodimerization of the drug target, leading to transactivation of the non-drug bound partner (132). Selective BRAF inhibitors have been developed such as vemurafenib, dabrafenib, and encorafenib with more potent inhibitory activity. However, these type I inhibitors lead to paradoxical ERK activation through allosteric activation of CRAF in tumors with class 2 or class 3 BRAF mutation (**Figure 2**).

Subsequent type II RAF inhibitors have been more successful in addressing these issues. These inhibitors stabilize RAF in its inactive conformation (“DFG out”), and, although they can also induce RAF dimerization, they bind concomitantly to both RAF partners and inhibit both protomers. Therefore, they are able to allay the issues of transactivation (11). To this effect, tovorafenib (DAY101), a type II pan-RAF inhibitor, has shown tumor inhibition in preclinical models and promising early clinical results in pLGG (133).

Despite these improvements, universal and durable response to MAPK pathway inhibition in pLGG has still not been achieved. Resistance can still occur, manifested by disease progression within months of commencing treatment. Resistance is mediated through several mechanisms. These include upstream activating mutations, downstream MAPK pathway alterations, activation of parallel signaling pathways, and increased expression of RTKs and BRAF amplification and alternative splicing (134). A significant adverse event observed in adults is hyperproliferative cutaneous events. These are mediated by BRAF inhibitor–induced paradoxical activation of MAPK pathway signaling in *BRAF* wild-type cells as described above.

In attempts to help combat resistance and provide alternative solutions, the MEK and, more recently, ERK signaling nodes have also been examined for their susceptibility to inhibition. This has been done both in isolation and in combination with other therapies. MEK inhibitors (MEKis) were particularly examined in pLGG harboring fusion mutations of BRAF, in an attempt to bypass the previously described paradoxical reaction to type I RAF inhibitors. Selumetinib (AZD6244), a MEKi, was shown to be effective in impairing cell viability in both *BRAF* V600E and *KIAA1549-BRAF* patient-derived xenograft pLGG models, an effect that was enhanced when used in combination with RAF inhibitors (135). This combination therapy currently represents the approach taken in melanoma to combat resistance, and the concept is currently being investigated in a phase II clinical trial combining dabrafenib and trametinib in *BRAF* V600–mutant pLGG (22). Last, inhibition of the ERK node provides an attractive target due to its role as the main effector in the MAPK pathway. It also directly interacts with RAF in a negative feedback loop that is possibly bypassing RAF and MEK inhibition. Recently, a novel agent ulixertinib has shown promise as an ERK inhibitor in patient-derived pLGG *in vivo* models, in isolation and when combined with MEKi (136). The subset of patients who might benefit with single agent rather than dual inhibitors is unknown. This will need to be further explored in early phase clinical trials.

### 4.2 ERK/MAPK pathway inhibition in pediatric low-grade gliomas

The success of MAPK pathway inhibitor therapies in the adult melanoma population generated interest in these agents in the neuro-oncology setting, particularly in pLGG, which nearly always harbor MAPK/ERK pathway oncogenic alterations. The frequency of MAPK pathway activation in this tumor type makes it a particularly attractive candidate to develop targeted therapies.

#### 4.2.1 Type I BRAF inhibitors

The first agent to be used as monotherapy in the pediatric solid tumor setting was the selective *BRAF* V600 inhibitor dabrafenib. This has been shown in phase I and II trials to have promising activity in relapsed or refractory *BRAF* V600–mutated pLGG, with overall response rate (ORR) of up to 80% across a collective cohort of 56 patients (12, 23, 137). The median times to first response averages 4 months, and the median duration of response (DOR) ranges from 11 to 26 months (12, 23, 137). Dabrafenib was well tolerated in most patients.

Monotherapy with the selective *BRAF* V600E inhibitor vemurafenib has also been shown to have efficacy in a phase I multi-center study of pediatric patients with recurrent or progressive *BRAF* V600E–mutant tumors (138). Of 19 patients treated for at least 12 months, only one patient had progressive disease (PD) on treatment. Vemurafenib was similarly well tolerated. A phase II study is ongoing (NCT01748149).

#### 4.2.2 MEK inhibitors

Given that the most common oncogenic MAPK pathway alteration in pLGG is the *KIAA-BRAF* fusion, in which upregulation of tumor activity is paradoxically observed with type I BRAF inhibition, downstream MEKis have been increasingly trialed over the last decade as an alternative agent both in these and other MAPK pathway–mutated tumors.

Selumetinib, a small-molecule potent inhibitor of MEK1/2, has been studied in several clinical trials. A phase I dose-finding trial in pediatric patients with recurrent or refractory LGG showed 20% of patients had a partial response, 80% of whom had confirmed *BRAF* aberrations (139). In the subsequent multicenter phase II trial, selumetinib was shown to have activity in patients with WHO grade I PA with either *KIAA1549-BRAF* fusion or *BRAF* V600E mutation, as well as with NF1-associated PA (140, 141). Of the 25 patients in stratum I, nine (36%) had partial response, nine (36%) had stable disease, and seven (28%) had PD (141). Selumetinib was well tolerated with the most common reported toxic effects being elevated creatine kinase (CK) or maculopapular rash (141).

These data suggest that selumetinib could be an alternative to standard chemotherapy with similar outcomes for these subgroups of patients. There are two phase III studies currently comparing standard chemotherapy to upfront selumetinib in patients with newly diagnosed pLGG in patients with/without NF1, respectively (NCT03871257 and NCT04166409).

Trametinib is another oral small-molecule MEK1/2 inhibitor that is currently being studied in phase I to III trials. The majority of current evidence is derived from case series that describe encouraging outcomes (108, 142). In the most recent multi-center retrospective study, of 18 patients with pLGG treated with trametinib for a variety of *KIAA1549-BRAF–, BRAF* V600E–, *FGFR1*-, or *NF1*-driven PD, 10 achieved stable disease, with two minor responses and six partial responses as best response (143). The minor and partial responses were observed in *KIAA1549-BRAF* fusion and NF1-associated cases. Median time to best response was 4 months (143).

Trametinib is also being studied prospectively in the pLGG setting. Interim data analysis in abstract forms from the first phase I/II trial of trametinib use in pediatric patients reports on 23 patients with *BRAF*-fusion LGG treated with trametinib (144). At time of interim analysis, trametinib had been well tolerated, with one confirmed partial response, and the majority of patients without PD (144). The current TRAM-01 trial is a phase II basket trial including four groups of progressive tumors (NF1-associated gliomas, NF1-associated plexiform neurofibromas, *KIAA1540-BRAF* fusion gliomas, and other MAPK-ERK pathway–activated gliomas), treated with trametinib monotherapy (NCT03363217). Interim analysis published in abstract form reported on 43 evaluable patients: four with partial responses, 18 with minor responses, 17 with stable disease, and four with progressive disease (145). Median time to response is 5.5 months, and median DOR is 6.1 months (145). Trametinib is also in phase II studies in Australia and New Zealand for NF1-associated OPG and plexiform neurofibromas (ACTRN12620001229965).

Binimetinib is another MEK1/2 inhibitor with good CNS penetration reported from a preclinical model that has been evaluated in a phase II trial for children with LGG and other MAPK pathway–activated tumors. The early published data from the non-NF1 non-surgical strata from 44 patients showed that 22 patients (50%) had either a minor or partial response (146).

#### 4.2.3 Combination therapy

With the improved PFS and OS observed in adult patients with metastatic melanoma treated with BRAFi + MEKi combination therapy compared with monotherapy, there has been a further investigation of the therapeutic potential of combination therapy in the setting of pediatric glioma.

Safety and efficacy results from the phase I/II trial recently published reports a cohort of 36 patients with previously treated pLGG and treated with dabrafenib and trametinib combination therapy (NCT02124772) (107). Median duration of exposure to combination therapy was 24 months (2.1–52.5). At the time of analysis, 89% of patients had stable disease or better per independent review using RANO criteria (147). The main adverse effects (AEs) observed were pyrexia and skin toxicity, with the majority reported as low grade (147). Importantly, objective response rates were higher for the combination therapy group than monotherapy group (25% vs. 15%) (107). Following these encouraging data, a phase II randomized study comparing first-line combination dabrafenib plus trametinib (D + T) versus traditional chemotherapeutic agents carboplatin plus vincristine (C + V) in *BRAF* V600–mutant–positive pediatric glioma patients has been undertaken (NCT02684058) (22). In the LGG cohort, patients with PD after surgery or non-surgical patients requiring systemic treatment were randomized 2:1 to receive D + T or C + V. There were 110 patients randomized in the pLGG cohort, and the median follow up time was 18.9 months. The ORR was 47% in the D + T group vs. 11% in the C + V group (p < 0.001). Median PFS was 20.1 months in the D + T group vs. 7.4 months in the C + V group (p < 0.001). Notably, toxicity was less in the D + T group, with grade 3 adverse events 47% vs. 94% in the C + V group, and there were fewer treatment discontinuations due to AEs (4% vs. 18% in the C + V group). This randomized study contributes encouraging results, suggesting that the combination BRAF and MEK inhibition may be an efficacious and well-tolerated upfront treatment strategy for this patient population.

#### 4.2.4 Type II pan-RAF kinase inhibitors

As described above, to avoid paradoxical activation of the MAPK pathway as described with type I BRAFi, type II RAF inhibitors have been developed (148). Two pan-RAF inhibitors, belvarafenib and tovorafenib, are currently undergoing assessment in open clinical trials.

Tovorafenib/DAY101 is an oral, brain-penetrant, highly selective type 2 pan-RAF inhibitor that does not result in paradoxical activation of the MAPK signaling pathway. A phase I trial in relapsed refractory low-grade gliomas with MAPK pathway alterations showed complete responses (CRs) (two of nine), partial responses (two of nine), and with stable disease (three of nine) (133). The interim results of the FIREFLY-1 study (NCT04775485), a phase II trial in RAF-altered pLGG, were recently released, reporting an objective response rate of 64% and clinical benefit (partial response and stable disease) in 91% of patients from the first 22 patients analyzed (149). The median time to response was 2.8 months in a heavily pre-treated population, with a median of three lines of therapy prior to enrolment. The majority of adverse events were grade 1 or 2 in nature, with most common side effects seen being rash, increase in blood creatinine, and hair color changes. Treatment-related grade 3 or greater toxicities occurred in 36% of patients (149). On the basis of these data, a randomized phase III trial testing upfront tovorafenib in pLGG is upcoming (NCT05566795).

### 4.3 ERK/MAPK pathway inhibition in pediatric high-grade gliomas

The majority of data reporting the use of MAPK pathway inhibitors in children is in the LGG setting, given their high prevalence. However, 5% to 10% of pediatric HGGs (pHGGs) harbor a mutation activating this pathway (91), mostly in *NF1* and *BRAF* genes.

Given the poor outcome of patients with high-grade glioma and with limited treatment options, developing targeted therapeutic strategies is critical. Single-agent dabrafenib has shown durable objective responses in many children with relapsed and refractory pHGG with *BRAF* V600E mutation (150).

A retrospective review of 19 pediatric patients with *BRAF* V600E–mutant HGG treated upfront with off-label BRAFi ± MEKi reports a 3-year PFS and a 3-year OS of 65% and 82%, respectively, which is improved compared to a historical control cohort treated with conventional therapies (151). On the basis of these encouraging data, upfront targeted therapy combining dabrafenib and trametinib after focal radiation for *BRAF* V600–mutant pHGG is being evaluated prospectively in a phase II clinical trial (NCT03919071). More recently, Hargrave and colleagues presented their study (NCT02684058) on dual BRAF and MEK inhibition in relapsed and refractory pHGG (152). This study is now closed to accrual. Forty-one patients with grade III/IV gliomas were enrolled and received dabrafenib and trametinib for a median time of 72.2 weeks. The ORR was 56.1%, with a median DOR of 22.2 months and median PFS of 9 months (152). In this population with otherwise dismal outcomes, they showed OS of 76.3% at 12 months, leading many neuro-oncologists to push for upfront management of children with these inhibitors. Although these studies clearly report the benefit of targeted therapy in pHGG with *BRAF* mutation in a tumor type mostly resistant to conventional approaches, the place of this therapy in an upfront setting is still to be proven and is yet to be approved by regulatory agencies for this indication. Clinical trials using these therapies upfront in this setting are underway internationally.

### 4.4 ERK/MAPK pathway inhibition in adult gliomas

*BRAF* V600 mutations have been identified in several adult glioma subtypes, including PXAs, gangliogliomas, anaplastic gangliogliomas, PAs, and, more rarely, adult HGGs (aHGGs) including GBM, with an overall incidence of 4% (153). Standard of treatment for adult glioma currently comprises surgery, chemoradiotherapy with TMZ. Lomustine and/or bevacizumab is a common salvage regimen at recurrence or progression. Because of the overall poor prognosis of *BRAF* V600–mutant aHGG or progressive aLGG, targeted therapy is an appealing approach for these patients.

Several case reports have described marked and often rapid responses of aHGGs to BRAF inhibitors and BRAF-MEKi combinations (154–157). In three cases of relapsed aHGG with widespread leptomeningeal disease, single-agent dabrafenib led to marked improvement within 2 months of starting therapy, the earliest within 1 week of initiation (155). One patient remained on dabrafenib after 27 months with ongoing complete radiologic response (155). Kushnirsky et al. described a case of multiply relapsed BRAF V600E–mutated GBM who was treated at first relapse with a BRAF inhibitor (PLX8394) with partial response (156). After further progression, the patient received dabrafenib and trametinib and eventually had a CR after 11 months on treatment. In addition, in one case of relapsed adult epithelioid GBM with widespread leptomeningeal dissemination, the patient demonstrated a complete metastatic radiological response after 4 weeks of treatment with combination dabrafenib and trametinib (157).

The above examples highlight an evolving role for the use of ERK/MAPK pathway inhibition in adult HGGs harboring BRAFV600 alterations, potentially representing an important therapeutic avenue for this traditionally treatment-resistant disease. In addition, the rapid responses described in these patients with widespread meningeal disease are suggestive of effective CNS penetration and drug delivery.

The use of BRAF and MEK inhibition in adult gliomas is also formally being investigated in several larger studies. The phase II VE-BASKET study includes single-agent vemurafenib in patients with recurrent *BRAF* V600E–mutant cancers including gliomas (25). The glioma cohort included 24 patients of various histologies, including LGG and HGG. Although some durable responses were seen, with median PFS of 5.5 months, most of the best responses were in the LGG group with a response rate of 43% (three of seven patients with PXA). In aHGG, the response rate was much lower at 9% (one of 11 patients) (25).

An ongoing large multicenter basket clinical trial has tested the combination of dabrafenib and trametinib in adults with recurrent or progressive *BRAF* V600–mutant gliomas (NCT02034110). Forty-five patients were enrolled in the HGG cohort resulting in an ORR of 33%, including three CRs and 12 partial responses with a median follow up of 12.7 months (29). The combination of binimetinib with encorafenib is currently being trialed in adults with *BRAF* V600–mutated HGGs (NCT03973918).

### 4.5 MAPK pathway alteration in craniopharyngioma

Craniopharyngiomas are rare brain tumors arising from epithelial remnants of the craniopharyngeal duct, typically in the suprasellar region. Previously defined as two subtypes, adamantinomatous craniopharyngioma (ACP) and papillary craniopharyngioma (PCP), these have been reclassified as their own distinct tumor types in the updated WHO 2021 classification (158). This is reflective of our evolving understanding of the distinct biology of each of these tumors. Although they are histologically benign entities with high OS, their treatment course is often complicated by significant morbidity and decreased quality of life. This is primarily due to disruption of the hypothalamic-pituitary axis but is also related to visual or cognitive impairment and vascular injury that occur either as a direct result of the location of their tumor or as complication of surgery and/or radiation treatment. As we have gained further molecular understanding of these tumors, therapeutic targets have been identified to help improve the current treatment paradigm.

ACPs are by far the most common type of craniopharyngioma and are found in both the pediatric and adult population. They are usually a mixed cystic/solid lesion and histologically characterized by palisading epithelium, wet keratin, and stellate reticulum. Studies have shown that these are driven by dysregulation of the Wnt pathway, typically *via* an exon 3 *CTNNB1* mutation (159). This leads to formation of B-catenin expressing cell clusters that are thought to play the critical role in tumorigenesis. Most recently, these clusters have been found to express several growth factors that activate the MAPK pathway, evidenced by identification of phosphorylated ERK1/2 at the leading edge of tissue invasion near these clusters (160). In murine and *ex vivo* cultures of ACP, inhibition of MAPK pathway with the MEKi trametinib correlated with reduction of phosphorylated ERK1/2 levels and, consequently, significantly reduced proliferation and increased apoptosis of tumor cells in the pre-clinical models (160). In addition, proteogenomics have shown that ACP groups with pLGG, interestingly in both *BRAF*-WT and *BRAF* V600E groups, further supporting that there is secondary upregulation of the MAPK pathway in ACP (161). Alongside this, an inflammatory milieu in ACP has been increasingly described, and the immune checkpoint protein CD47 has also been shown to activate the MAPK pathway in ACP cells (162).

Clinically, a single case report of a highly refractory young adult patient with ACP showed a significant, durable radiological response to binimetinib, a MEKi (163). Given the growing body of evidence to suggest a role for MAPK pathway in ACP, a phase II clinical trial is underway with the goal of evaluating RAF inhibition by tovorafenib in pediatric ACPs either in isolation or in combination with the immune checkpoint inhibitor nivolumab (NCT05465174).

In contrast, PCPs represent only approximately 10% of craniopharyngiomas and are almost exclusively seen in the adult population. These lesions are predominantly solid with well differentiated nonkeritanizing squamous epithelium and papillary fibrovascular stroma. Their molecular profile is similar to that of pLGG in that they are molecularly bland, with a low mutation burden, and are characterized by mutant *BRAF*. The role of *BRAF* as a driver in PCPs was first identified by Bratsianos et al., who, through WES, identified recurrent somatic mutations in *BRAF* V600E in 100% of their samples (three patients) (164). This was further interrogated through targeted genotyping and Immunohistochemistry (IHC) across a broader population group. The prevalence of *BRAF* V600E mutations was found to be 94.4% (34 of 36 patients) (164). The exact mechanism of tumorigenesis in craniopharyngiomas is unconfirmed but has been suggested to be related to sustained proliferation and impaired differentiation of pituitary SOX2-positive cells, resulting from MAPK pathway activation (165). Despite the common upregulation of the MAPK pathway, these tumors have been shown to have separate protein expression and methylation profiles from ACPs that further highlight their unique molecular profiles (166, 167).

Given the finding of *BRAF* V600E as a defining mutation in PCPs, there are multiple published case reports of their successful use both as a neoadjuvant tool and in relapsed/refractory disease (168). A case series of six patients showed promising responses, ranging from partial to CR in both heavily pre-treated cases and a patient that had only undergone biopsy (169). Following this, a phase II trial (Alliance A071601), which examined positively screened BRAF patients who were radiation naïve, showed that all 15 patients that were able to complete one course of combination therapy of vemurafenib + cobimetinib had an objective response (170). Given these findings, there have been multiple algorithms proposed as to how to incorporate BRAF inhibitors into current clinical practice either as a salvage in relapsed disease or as a neoadjuvant tool to achieve GTR with limited morbidity (168).

Current open trials targeting the MAPK signaling pathway in craniopharyngiomas include the following: a phase I trial assessing the combination of oral dabrafenib and trametinib in patients with BRAF-mutated PCP (NCT05525273); a phase II trial assessing the efficacy of vemurafenib and cobimetinib in *BRAF* V600E–mutated PCP (NCT03224767); the PNOC029 trial that will assess the tolerability and efficacy of combination therapy with PD-1 (nivolumab) and pan-RAF-kinase (tovorafenib) inhibition for newly diagnosed or recurrent craniopharyngioma, regardless of its subtype, in children and young adults (NCT05465174); and an upcoming phase II trial for pediatric APC assessing binimetinib (NCT05286788).

Overall, these findings highlight the role of the MAPK pathway not only in diagnosis but also in the treatment approach for craniopharyngiomas despite their unique biological profiles. More evidence will be required to confirm the hypothesis that medical therapy is able to significantly alleviate the long-term morbidity of the disease. As with the role of targeted therapies elsewhere, the decision of when to commence treatment and for how long remains unanswered and requires ongoing exploration in clinical studies.

### 4.6 Limitations of ERK/MAPK pathway inhibitors by the blood–brain barrier

Although the BBB plays a critical role in protecting the CNS from endogenous and exogenous toxins, this defense mechanism has long posed difficulty in ensuring adequate drug delivery to brain tissues. Developing targeted therapies that are able to effectively penetrate this barrier is a crucial element in drug design and trial evaluation to help achieve improved outcomes for CNS malignancies.

The BBB is a highly specialized interface of the brain microvasculature that is predominantly formed by brain endothelial cells. These cells are connected by tight junctions that allow them to maintain rigid control over the movement of ions and molecules from the blood to the brain. These cells are supported by astrocytes and pericytes that play an important role in regulation of the system with neuronal input. This physical barrier at the vessel interface is enhanced by efflux transporters that are embedded within the endothelial cells and can actively reduce drug distribution in the brain despite drug permeability (171). Notably, the key transporters P-glycoprotein and breast cancer resistance protein have been specifically implicated in export of anticancer therapy, including the BRAF/MEKis vemurafenib, dabrafenib, and trametinib (172, 173). Importantly, the BBB is not an immutable entity and undergoes significant changes in response to pathologies affecting the CNS. In presence of a brain tumor, the BBB is preferentially referred as the blood–brain tumor barrier (BBTB). Inflammatory changes, neoangiogenesis, or impairment of the blood flow by compression of existing vessels by the tumor, along with others alterations, increases the leakiness of the BBTB, however, in a highly heterogenous pattern. This explains why, despite the described active export of BRAF/MEKis, these medications have been shown to have good, albeit varied, clinical efficacy in MAPK pathway–altered intracranial melanoma metastases and primary brain tumors (147, 174). Whether this varied pattern of clinical efficacy is related to penetrance, tumor resistance or an interplay between them requires further exploration.

In this context, there has been concerted effort in developing other small-molecule inhibitors that more effectively cross the BBB. For example, the type II RAF inhibitor tovorafenib has been shown to have superior blood–brain penetrance in mice when compared with dabrafenib (175). Most recently, a medicinal chemistry approach reducing the molecular size and adding a carboxylic acid to help transform dabrafenib into a highly BBB penetrant molecule, named everafenib, resulted in superior treatment efficacy in mouse models of metastatic melanoma (176). Moving forward, it will be important to monitor the clinical efficacy of more penetrant agents and whether this is a key factor in their superiority. Specifically examining their impact on tumors that may not exhibit BBTB breakdown will also be telling.

## 5 Controversies and concerns of long-term use of BRAF inhibitors

### 5.1 Adverse effects

#### 5.1.1 Adverse effects of selective BRAF inhibitors

Emerging studies have demonstrated the safety and manageable side effect profile of BRAFi in children and adults. In general, these agents are well tolerated. Drug class toxicities for these agents include pyrexia, arthralgia, fatigue, headache, cutaneous toxicities and proliferative skin, and gastrointestinal disorders (177). AEs reported in studies of patients with glioma, summarized in **Table 2**, are similar to those in the broader literature, where the largest body of evidence comes from metastatic melanoma. Hargrave et al. (23) found fatigue (34%), cutaneous effects (rash (31%), dry skin (28%), and pyrexia (28%) as the most common AEs in 29 patients with *BRAF* V600–mutant pLGG treated with dabrafenib. Only two patients (6%) discontinued the treatment due to AEs. In another multinational study of BRAFi in *BRAF* V600E–mutated pediatric gliomas reported by Nobre et al. (137), 23% of the 67 patients enrolled required dose reduction or temporary discontinuation of drugs due to AEs. These AEs were mainly skin toxicities (n = 11, 16%). Overall, only three patients (5%) discontinued treatment altogether (rash, n = 1; hepatotoxicity, n = 1; benign transient melanotic lesions, n = 1).

**Table 2:** Adverse events of BRAFi and MEKi monotherapy reported in glioma trials.

BRAFi						MEKi					
Dabrafenib (23)			Vemurafenib (25)			Selumetinib (141)			Trametinib (143)		
Toxicity	Any (%)	Grade >=3	Toxicity	Any (%)	Grade >=3	Toxicity	Grades 1–2 (%)	Grade >=3	Toxicity	Any (%)	Grade >=3
Fatigue	34	0	Arthralgia	67	0	CK elevated	68	10	Any	89	44
Rash	31	0	Melanocytic nevus	38	0	Hypoalbuminaemia	62	0	Rash	38	11
Dry skin	28	0	PPES	38	0	Dyspnea	60	0	Paronychia	38	6
Pyrexia	28	0	Alopecia	33	0	Duodenal ulcer	58	0	Xerodermia	22	0
Rash maculopapular	28	9	Fatigue	29	4	Rash	58	10	Diarrhea	11	0
Arthralgia	25	3	Pruritis	29	0	Dry skin	56	0	Dizziness	11	0
Headache	22	0	Rash	29	13	Fatigue	56	0	Eczema	11	6
Vomiting	22	0	Folliculitis	25	0	Anemia	56	0	Fatigue	11	0
Arthralgia	3	3	Headache	25	0	Diarrhea	54	4	Oral mucositis	11	0
DIC	3	3	Constipation	21	0	Vomiting	44	0	Left ventricular dysfunction	6	0
EF decreased	3	0	Diarrhea	21	0	Paronychia	38	6	Pancreatitis	6	6
Hypotension	3	3	Nausea	21	0	EF decreased	38	2	Constipation	6	0

CK, creatinine phosphokinase; DIC, disseminated intravascular coagulation; EF, ejection fraction; PPES, palmar-planar erythrodysesthesia.

In another phase I study testing dabrafenib in 27 children and adolescent patients with *BRAF* V600–mutated solid tumors (12), almost all patients experienced at least one AE (n = 26, 96%), and six patients (22%) reported grade 3 or 4 AEs considered as related to the study drug. The most common AEs were fatigue (33%), vomiting (30%), headache (26%), and hypophosphatemia (26%), whereas the most common grade 3 or 4 AEs were arthralgia and maculopapular rash (each n = 2, 7%). No patients discontinued treatment on account of study drug-related AEs. No patients developed cutaneous squamous cell carcinoma (SCC) as has been reported in adults, and there are no reports of secondary malignancy related to dabrafenib, although the follow up period was only 3 years.

Vemurafenib monotherapy side effects appear to be proportional to dose and length of exposure to the drug (177). In the phase II VE-BASKET trial, vemurafenib was associated with arthralgia (67%), rash (42%), palmar-planter dysesthesia (38%), fatigue (33%), other cutaneous toxicities, and gastrointestinal disorders such as nausea (21%) and diarrhea (21%) (25). Vemurafenib is also associated with ultraviolet A-photosensitivity (41%) (178), although this is considered to be related to the chemical structure of the drug rather than its BRAF-inhibiting activity (177, 179, 180). When combined with MEKi cobimetinib, the incidence of photosensitivity reduces however still remains as high as 34% (177).

As mentioned above, encorafenib is being trialed in combination with binimetinib in NCT03973918. Data from studies of melanoma patients demonstrate that encorafenib monotherapy results in hyperkeratosis (59%), alopecia (56%), palmoplantar erythrodysesthesia (52%), fatigue (44%), arthralgia (43%), nausea (38%), and myalgia (36%) (177, 180). In adults, encorafenib has been reported in up to 8% of patients to cause transient facial nerve palsy (177).

#### 5.1.2 Adverse effects of MEKi

Drug class toxicities of MEKi often occur early in treatment and are alleviated with time. These include fatigue, anemia, cutaneous toxicities, gastrointestinal toxicities, liver transaminase elevation, ocular toxicities, muscular problems, and cardiovascular toxicity (177).

In the study of Selt et al., trametinib monotherapy of 18 patients with pLGG resulted in maculopapular rash (38%), paronychia (38%), acneiform rash (28%), and xerodermia (22%) with six patients (33%) requiring dose reduction and two patients (11%) discontinuing treatment due to acneiform rash (143). For patients enrolled in strata 1 and 3 of NCT01089101 described above, selumetinib monotherapy resulted most commonly in creatine phosphokinase elevation (68%), hypoalbuminemia (62%), dyspnea (60%), diarrhea (58%), duodenal ulcer (58%), dry skin (56%), anemia (56%), and rash [acneiform (62%) and maculopapular (62%)] (141). Nineteen of 50 patients (38%) required dose reduction, and five patients (10%) discontinued the drug due to toxic effects. No deaths were reported.

Although early reports suggest that binimetinib is active with a manageable toxicity profile in children with pLGG, 11 of the 57 patients (19%) discontinued the drug in the first year due to toxicity and an additional 27 patients (47%) required dose reduction (146). Grade 3 and 4 toxicities were reported as CK elevation (19%), rash (14%), truncal weakness (14%), and transient colitis (2%) (146).

#### 5.1.3 Adverse effects of combination BRAFi/MEKi

In adults, the combination of BRAFi and MEKi significantly reduces paradoxical activation of alternate MAPK pathway. This also results in reduced skin toxicity but increased pyrexia, gastrointestinal, and ocular toxicities (181). More recent trials have evaluated the effects of combining dabrafenib and trametinib in combination in *BRAF* V600–mutant pLGG (107). Pyrexia (50%), dry skin (42%), dermatitis acneiform (39%), and fatigue (39%) were frequent AEs. However, most AEs were grade 1 and manageable, with eight patients (22%) withdrawing/discontinuing treatment because of AEs. In NCT02684058 described above, comparing first-line dabrafenib + trametinib with standard of care chemotherapy in pLGG, those in the experimental arm reported fewer treatment-related grade 3 or higher AEs [n = 19 (26%) vs. n = 29 (88%)] (22). Furthermore, the experimental arm reported less AEs, resulting in discontinuation of therapy [n = 2 (3%) vs. n = 3 (9%)]. The most frequent reported AEs of dabrafenib and trametinib in combination are highlighted in **Table 3**.

**Table 3:** Adverse events of combination therapy reported in glioma trials.

Dabrafenib + Trametinib (22)			Dabrafenib + Trametinib (29)		
Toxicity	Any (%)	Grade >=3 (%)	Toxicity	Grades 1–2 (%)	Grade >=3 (%)
Any	100	47	Any	92	53
Pyrexia	68	8	Fatigue	41	9
Headache	47	1	Headache	38	5
Vomiting	34	1	Nausea	33	2
Fatigue	32	0	Pyrexia	31	2
Diarrhea	29	0	Rash	29	0
Dry skin	26	0	Vomiting	26	2
Nausea	25	0	Anemia	22	0
Anemia	15	0	Constipation	21	0
ALT increased	14	5	Arthralgia	21	2
Neutropenia	14	10	AST/ALT increased	17	3
Neutrophil count decreased	14	5	Myalgia	14	2
Constipation	12	0	Diarrhea	14	2
WBC count decreased	11	0	Neutropenia	10	5
Platelet count decreased	5	0	Ejection fraction decreased	10	2

Importantly, the AEs of BRAFi/MEKi are generally well tolerated. Pyrexia events are usually episodic and occur during the first month of treatment. Symptoms resolve with dose reduction and/or interruption and supportive treatment (26). In the adult population, guidelines exist for the management of skin toxicities (12). Similar guidelines exist as part of the Children’s Oncology Group ACNS1723 protocol (NCT03919071) mentioned above. The most common skin toxicities include rash, which may be treated with emollients, antihistamines, and analgesia. Topical steroids are occasionally necessary. Photosensitivity reactions can be mitigated through patient education and the use of sunscreen and UV-protective clothing (26). Liver enzymes should be measured at baseline and monitored regularly throughout the course of treatment with dose reductions and/or interruptions as indicated for persistent or recurrent grade 2 or grade 3 liver enzyme derangement (26).

The observed toxicities in pediatric studies are similar to those reported in the adult population using BRAFi, aside from a notable absence of proliferative skin and gastrointestinal toxicities (181, 182). This is likely due to a combination of factors, including age and predisposing risk factors (181). For example, benign naevi are reported in children but without malignant progression to keratoacanthoma and SCC that has been reported in adults (183, 184). The vast majority of SCCs reported in adults taking BRAFi occurred in chronically sun-damaged skin (184). Furthermore, secondary melanomas, gastric and colonic polyps, and recurrences of pre-existing malignancies have been reported in adult patients (184). The development of secondary malignancies does warrant close attention, particularly given that the optimal duration of treatment in pediatric patients is currently unknown.

Cardiovascular adverse events associated with BRAFi and MEKi have been reported in several adult studies (185). The underlying pathophysiology is thought to be the interference of cardiovascular MAPK signaling, generating oxidative stress and apoptosis of myocytes as well as impaired angiogenesis (185). This results in cardiomyopathy with decreased cardiac ejection fraction. Cardiac arrhythmia resulting from QTc interval prolongation has also been reported in adults with predisposing factors such as hypertension and ischemic heart disease (181). Although these toxicities may be less prevalent in childhood due to absence of predisposing factors, long-term effects on the cardiovascular system are unknown in children.

In more recent pediatric studies, electrolyte disturbances have been reported including hypo- and hypernatremia (107). Therefore, in patients with hypothalamic or suprasellar tumor involvement, a history of diabetes insipidus or other electrolyte disturbance sodium levels should be monitored closely.

In light of the known AEs, prior to commencement of BRAFi ± MEKi therapy, it is important to perform dermatologic and ophthalmologic examination, electrocardiogram (ECG), measurement of blood pressure, serum electrolytes, and liver function tests (181). Furthermore, as BRAFis are metabolized by the CYP450 system, it is important to consider drug interactions and altered metabolism when used in combination with other CYP450 metabolized drugs.

The modern era of targeted therapy heralds a new phase in treatment of childhood cancer. Novel agents offer the promise of more effective treatment while sparing toxicities. However, targeted agents have the potential to demonstrate off-target effects. Studies in both adult and pediatric settings have outlined short term AEs of BRAFi and MEKi alone and in combination. The long term effects of these agents are unknown and will warrant close attention.

### 5.2 Duration of therapy

Despite the promising response to BRAFi ± MEKi therapy demonstrated in pediatric gliomas, little is known regarding the optimal duration of therapy. In 13 patients with BRAF V600–mutant glioma treated with trametinib the estimated 24-month DOR rate was 100% for monotherapy and a median DOR of 33.6 months [95% CI, 11.2 to not reached (NR)] and estimated 24-month DOR rate of 80% (95% CI, 20–100) for those with dabrafenib and trametinib combination therapy (107). Nobre et al. reported an 80% response rate in 69 children with pediatric gliomas treated with dabrafenib or vemurafenib (137). Of these children, most (n = 48, 86%) with pLGG had sustained response with median treatment time of 17.4 months (range of 6–61 months). However, of the 17 patients who discontinued therapy, 76.5% (n = 13) of tumors had rapid regrowth (median of 2.3 months). When rechallenged with BRAFi alone or in combination with MEKi, 90% of those patients responded. These data suggest that long-term use of BRAFi alone or with addition of MEKi is required in this setting. In the study of Manoharan et al. of patients with recurrent/progressive pLGG treated with trametinib, in which four patients were identified with *KIAA1549-BRAF* fusion, median time to best radiological response was 9.8 months, with one patient in the cohort achieving best radiological response at 22 months, suggesting the need for protracted treatment courses (108). However, there are limited data regarding long-term use of BRAFi and or MEKi, including AEs and developmental outcomes.

Most data about disease response following discontinuation of BRAFi therapy are derived from adults with BRAF V600–mutant melanoma. Stege et al. (186) reported on 37 patients retrospectively selected through the multi-center skin cancer registry ADOReg who had achieved CR with BRAFi alone or in combination with MEKi as first-line therapy. Median duration of therapy was 16 months, with 11 patients (30%) still receiving treatment at data cutoff. Common causes of discontinuation were PD (n = 13, 50%) and toxicity (n = 6, 23%). PD was found in 22 (59%) overall and was most common in patients who discontinued treatment (n = 13/22, 59%). Of those patients who progressed following discontinuation, 60% responded to rechallenge. Patients with durable CR after treatment cessation were predominantly those who received treatment for longer than 12 months, who discontinued treatment for reasons other than PD or toxicity and who discontinued treatment for longer than 6 months.

Di Guardo et al. (187) reported on 24 patients at a single institution with BRAF V600–positive metastatic melanoma who discontinued BRAFi ± MEKi for reasons other than PD. Reasons for discontinuation were unacceptable toxicity (n = 19, 79%) and withdrawal of consent (n = 5, 21%), at which time 17 (71%) were in CR and 7 (29%) were in PR. At a median follow up time of 37.8 months, PFS at 12 months was 70.8% and at 24 months was 58.3%. Similarly, Bédouelle et al. (188) reported on 29 patients at a single center with BRAF-mutated melanoma, treated with BRAFi ± MEKi. Median treatment duration was 9.7 months, with relapse rate following discontinuation of 69% at 12 months and 76% at 36 months. After relapse, 53% of patients responded to rechallenge with BRAFi ± MEKi. In summary, risk of disease progression following cessation of treatment is high in adult patients with BRAF V600–positive melanoma. Whether this outcome translates to patients with pLGG using BRAFi ± MEKi is unknown although as discussed above, many do respond on treatment reinitiation.

Furthermore, given the long-term use of these medications, longitudinal efficacy and toxicity data are needed in children. The effects on growth, development, and long-term disease control given the balances between oncogene senescence and growth in glioma are to date unknown. In addition, it remains completely unknown in which patients we can stop and trial off-drug and those who we must continue on therapy. Many collaborative teams around the world are actively seeking to answer these questions prospectively.

## 6 The Australian and New Zealand experience

### 6.1 Current Australian and New Zealand approvals for ERK/MAPK inhibitors

In Australia, there are currently three BRAFis approved for use by the Therapeutic Goods Administration (TGA). Vemurafenib was first approved in April 2012 for the treatment of unresectable Stage IIIc or Stage IV metastatic melanoma positive for *BRAF* V600–mutant kinase activity (189). Dabrafenib mesilate was approved in August 2013 for the treatment of patients with *BRAF* V600 mutation–positive unresectable stage III or stage IV melanoma (190), with approval of its use in combination with the reversible allosteric inhibitor of MEK (MEK1 and MEK2) trametinib shortly following in 2014 (191). More recently, encorafenib was approved in 2019 in combination with binimetinib for patients with unresectable or metastatic *BRAF* V600 mutation–positive melanoma and in combination with cetuximab for patients with metastatic colorectal cancer with *BRAF* V600E mutations (192). To date, there remains no currently approved BRAFi for glioma in any age group or tumor grade.

In New Zealand, Medsafe is the New Zealand Medicines and Medical Devices Safety Authority, responsible for the regulation of therapeutic products, and is the equivalent of the TGA. Both dabrafenib and vemurafenib monotherapy as well as dabrafenib/trametinib and vemurafenib/cobimetinib combination therapies are approved indications for unresectable stage III or metastatic melanoma that is *BRAF* V600E–mutated. In addition, the dabrafenib/trametinib combination therapy is also approved for locally advanced/metastatic anaplastic thyroid cancer and advanced NSCLC harboring a *BRAF* V600 mutation. Like Australia, approvals for their use in glioma remain lacking.

### 6.2 Current access to BRAF inhibitors: Trials/managed access programs/compassionate access

In Australia, although TGA approval exists for BRAFi, access to these medications for children and adults affected by *BRAF*-altered glioma relies on enrolment into clinical trials, company-specific Managed Access Programs, hospital-specific drug usage committees, and through private funding, particularly in the AYA and adult setting. Unapproved medications can be prescribed in Australia by applying to a TGA Special Access Scheme (193). Because of the substantial cost of these drugs [nearly $9,000 AUD for 1 month of dabrafenib, for example, see (194)], private funding is not accessible to every patient setting up a system of inequality to care. Clinical trials in Australia can supply unapproved medications *via* an expeditious clinical trial notification (CTN) scheme (195), instead of the necessity of investigational new drug agreements. In Australia, there is currently access to vemurafenib/cobimetinib for adult patients with *BRAF*-mutant cancers *via* a national clinical trial platform called the Molecular Screening and Therapeutics Program (MoST): Addendum 12, Substudies 27-30 (ACTRN12620000861954).

Following an Australian Parliamentary inquiry into approval processes for new drugs and novel medical technologies under the direction of the former Health Minister of Australia, the Standing Committee on Health, Aged Care and Sport, issued a position statement “The New Frontier – Delivering better health to all Australians” (196). Several cancer advocacy groups were consulted, including the Australian and New Zealand Children’s Haematology/Oncology Group (ANZCHOG) and the Medical Oncology Group of Australia (MOGA). The ANZCHOG Australian Cancer Plan Submission endeavors to enhance access for pediatric cancer patients to promising novel agents outside clinical trials (196). In this submission, recommendations relevant to children with cancer included the following:

- sponsor submission fee waivers to encourage registration of orphan drugs;
- alterations of the TGA’s Orphan Drugs Program to specifically treat pediatric patient populations as distinct from their adult counterparts;
- the repurposing of existing medicines to treat alternative conditions;
- a range of recommendations on improving the clinical trial system in Australia.

Included in the 2020 parliamentary enquiry is the recommendation for molecular indications (Recommendation 13), which endorses the reform of regulatory and reimbursement processes to enable therapeutic goods to be registered and reimbursed by molecular indication (196).

From these hearings, several recommendations were made, including that the assessment process for the Life Saving Drugs Program (LSDP) be streamlined and delays in access to treatments be reduced by ensuring that a sponsor only need lodge one application for one Health Technology Assessment pathway. The Committee recommended either of the following:

- providing sponsors with an immediate pathway to the LSDP Expert Panel (instead of waiting for a Pharmaceutical Benefits Advisory Committee (PBAC) determination), or
- providing a pathway by adjusting the Pharmaceutical Benefits Scheme (PBS) section 100 program, with specific criteria, as with other section 100 programs.

The report highlights the importance of ensuring that the LSDP will integrate with an increasing number of precision medicine applications into the future.

As only recently released, few changes have been seen to speed drug access to new and novel drugs in Australia. However, much hope is derived from these announcements that faster, easier, and equitable access to new and novel medications including MAPK inhibitors will occur in the near future.

Pharmac is the New Zealand Government Authority responsible for consultation and decision-making on the funding of medications within a fixed budget. Although the above indications are all approved, they are not yet funded for these indications.

Access to precision medication through clinical trial enrolment is limited in New Zealand and dependent on currently open clinical trials. At present, patients with NF1 with recurrent or refractory OPG are eligible to receive trametinib through participation in the TiNT trial (ACTRN126200001229965). Patients with newly diagnosed RAF-altered glioma may also be eligible in the future for targeted treatment. There remains a significant gap in precision medicine access for children with both CNS and non-CNS tumors with RAF alteration through clinical trial or compassionate access.

Fortunately, within the two Pediatric Oncology/Hematology services in New Zealand, there are existing funding avenues for public hospitals to give pharmaceuticals including BRAFi for the treatment of cancer. The prescription of precision medicines for the molecular indications outside of their approved use is facilitated by the National Child Cancer Network (NCCN) consensus guidelines, for which medications meet this threshold, and relies on two processes. Initially, endorsement to use a precision agent (BRAFi or MEKi) occurs by multidisciplinary meeting discussion. Following that, prescription is by a registered medical practitioner under section 29 of the Medicine Act.

If sufficient clinical data exist to support the use of these agents, then pediatric patients with MAPK pathway alterations as drivers of a cancer or tumor can use a specialized funding pathway to ensure equity of access to these agents for all children in New Zealand. Given the current state of recently closed and ongoing clinical trials, these agents are not usually prescribed in the first-line setting, although as greater information is reported in randomized studies against current conventional chemotherapy standards of care, this is likely to change.

### 6.3 Perspective for rare cancers: Mechanism of action-based drug approval

Advances in precision oncology have allowed for an alternative to the traditional organ-specific treatment approaches. Mechanism of action-based approval allows a tissue- and histology-agnostic method through targeting specific biomarkers and genetic alterations irrespective of tumor location (197). In 2017, pembrolizumab gained FDA approval for the treatment of microsatellite instability-high solid tumors, regardless of the primary site (198). This represented the first FDA approval of a cancer treatment for an indication using a common biomarker.

Two further agents to receive mechanistic-based FDA approvals are larotrectinib and entrectinib, in 2018 and 2019, respectively, targeting *NTRK* gene fusions tumors in adult and pediatric populations (198, 199). Continuing from this in 2020, the TGA granted larotrectinib provisional tissue-agnostic approval in Australia for indications in alignment with FDA approvals (200).

Early in the genomics era, the prevalence of *BRAF* V600 mutations was reported in 43 tumor types across 2963 samples in an AACR GENIE database (201). The potential existed for targeted, mechanism of action-based therapy in this population. However, pursuit of tissue-agnostic approvals in BRAFi was dissuaded by a phase II pilot study of vemurafenib in patients with *BRAF* V600E–mutant colorectal cancer that did not show meaningful clinical activity (202). The rapid feedback activation through EGFR pathways caused by BRAF inhibition in colorectal cancer was subsequently overcome by the addition of anti-EGFR agents, resulting in significant improvements to OS and ORR (10). Therefore, although tissue-agnostic approvals will hopefully improve access, caution must be exerted on drug use without excellent preclinical or clinical evidence in patients where alternative therapies exist.

### 6.4 Drug access outside clinical trials

Although, ideally, all patients are enrolled on clinical trials, the sheer volume of mutations, drugs, and combinations that may be targetable limits the number of clinical trials that may be opened at any one center. As such, there is a heavy reliance on off-label use of drugs. However, appetite for risk of off-label use of drugs differs at different treating centers, again resulting in equity/access issues for patients nationally (196). In their submission to the parliamentary inquiry, MOGA submitted that “the different coverage of on-label and off-label indications in hospital and PBS formularies may affect the continuity and affordability of treatment for patients.” (196) Harmonization of drug approvals and access through a national formulary would allow easier and equitable access to all patients.

For those diseases with large volumes of patients where clinical trials may be more relevant, the Standing Committee on Health, Aged Care, and Sport in Australia recommended that all levels of government prioritize and implement with urgency the harmonization of Human Research Ethics Committee (HREC) and Site-Specific Assessment submissions into one Australian online platform and enable parallel review by HRECs and Research Governance Offices to improve access to and enrollments in clinical trials. They suggest the following:

- The platform should be developed within the purview of the Australian Commission on Safety and Quality in Health Care.
- This work should be a continuation from the work prepared as part of the National Clinical Trials Governance Framework.

Although many of the findings to date have yet to be implemented, the acceptance and feedback from clinicians and patients in Australia who are actively seeking easier and more affordable access to novel therapies give much excitement and hope to neuro-oncology care now and into the future.

## 7 Conclusions

The discovery of molecular drivers in both pediatric and adult gliomas heralds a new exciting phase in the treatment of these cancers. Alterations in MAPK/ERK signaling pathway offer opportunities for druggable targets and, hence, an explosion in recent years of research in this field. As detailed in this review, there are promising data to support the use of RAF inhibitors both as monotherapy and in combination for patients with glioma. Multiple challenges remain, including access to therapy, optimal duration of treatment, toxicity management, and the impact of targeted treatments on natural history of disease.

## Author contributions

D-AK-Q, JRH, and JW contributed to the conception and design of the review. ST, CM, PP, and MT wrote the first draft of the manuscript. D-AK-Q, JRH, JW, BC, SLa, KT, and AD wrote sections of the manuscript. All authors contributed to the article and approved the submitted version.
